# Carbohydrate-binding modules (CBMs) revisited: reduced amount of water counterbalances the need for CBMs

**DOI:** 10.1186/1754-6834-6-30

**Published:** 2013-02-26

**Authors:** Anikó Várnai, Matti Siika-aho, Liisa Viikari

**Affiliations:** 1 Department of Food and Environmental Sciences, University of Helsinki, P.O. Box 27, 00014, Helsinki, Finland; 2 VTT Technical Research Centre of Finland, P.O. Box 1000, 02044 VTT, Espoo, Finland

## Abstract

**Background:**

A vast number of organisms are known to produce structurally diversified cellulases capable of degrading cellulose, the most abundant biopolymer on earth. The generally accepted paradigm is that the carbohydrate-binding modules (CBMs) of cellulases are required for efficient saccharification of insoluble substrates. Based on sequence data, surprisingly more than 60% of the cellulases identified lack carbohydrate-binding modules or alternative protein structures linked to cellulases (dockerins). This finding poses the question about the role of the CBMs: why would most cellulases lack CBMs, if they are necessary for the efficient hydrolysis of cellulose?

**Results:**

The advantage of CBMs, which increase the affinity of cellulases to substrates, was found to be diminished by reducing the amount of water in the hydrolytic system, which increases the probability of enzyme-substrate interaction. At low substrate concentration (1% w/w), CBMs were found to be more important in the catalytic performance of the cellobiohydrolases TrCel7A and TrCel6A of *Trichoderma reesei* as compared to that of the endoglucanases TrCel5A and TrCel7B. Increasing the substrate concentration while maintaining the enzyme-to-substrate ratio enhanced adsorption of TrCel7A, independent of the presence of the CBM. At 20% (w/w) substrate concentration, the hydrolytic performance of cellulases without CBMs caught up with that of cellulases with CBMs. This phenomenon was more noticeable on the lignin-containing pretreated wheat straw as compared to the cellulosic Avicel, presumably due to unproductive adsorption of enzymes to lignin.

**Conclusions:**

Here we propose that the water content in the natural environments of carbohydrate-degrading organisms might have led to the evolution of various substrate-binding structures. In addition, some well recognized problems of economical saccharification such as unproductive binding of cellulases, which reduces the hydrolysis rate and prevents recycling of enzymes, could be partially overcome by omitting CBMs. This finding could help solve bottlenecks of enzymatic hydrolysis of lignocelluloses and speed up commercialization of second generation bioethanol.

## Background

Enzymatic hydrolysis of lignocellulosic plant cell walls to platform sugars is a fundamental process with considerable industrial importance, currently approaching commercialization. Several demonstration plants have been established around the world to convert lignocellulosic raw materials to ethanol, which is expected to be commercialized soon, partially replacing first generation ethanol during this decade. The production of cellulosic ethanol, however, is still uneconomical without governmental subsidies to reduce the price of cellulosic ethanol to the price level of regular gasoline [1]. Despite the 20-fold reduction in enzyme production costs reported by the major enzyme producing companies during the last decade, enzymes still make up at least 15% of ethanol production costs [2,3]. Further reduction of saccharification costs is thus necessary and could potentially be achieved by recycling enzymes.

A spectrum of well-characterized enzymes is needed for efficient saccharification of cellulose: cellobiohydrolases (CBHs, EC 3.2.1.91 and 3.2.1.176) for hydrolyzing the glycosidic linkages, mainly on the crystalline regions of cellulose and releasing cellobiose units from the reducing and non-reducing chain ends; endoglucanases (EGs, EC 3.2.1.4) for cleaving cellulose chains in the amorphous regions; the recently discovered oxidoreductases, which contribute to hydrolysis via oxidative cleavage of cellulose [4] and finally β-glucosidases (EC 3.2.1.21), for producing glucose from the solubilized cello-oligomers and dimers. In particular, CBHs have been traditionally considered the main enzymes contributing to the hydrolysis of crystalline cellulose by fungi, forming approximately 80% of total secreted protein, *e.g.,* in *Trichoderma reesei*, the most thoroughly studied fungus for the production of cellulases. The majority of fungal CBHs belong to the glycoside hydrolase families GH7 (CBH I) and GH6 (CBH II), based on their sequence similarity and predicted structural and functional relationships [5].

Traditionally, the structure of plant cell wall degrading systems has been distinguished among aerobic (mostly fungi) and anaerobic microbes (mostly bacteria). In most of the aerobic organisms, various cellulases and other plant cell wall degrading enzymes are secreted extracellularly and play extensive biochemical synergy. Cellulases of these organisms typically have a two-domain structure containing a catalytic or core domain, where the catalysis takes place, and a carbohydrate binding module (CBM). In contrast, most of the anaerobic microorganisms are recognized to produce an array of hydrolytic enzymes associated with the integrating subunit (scaffoldin), containing the CBM and forming cell-bound supramolecular complexes, cellulosomes [6]. It is, however, difficult to make a clear distinction between the microorganisms based on their cell wall-degrading enzyme systems as fungi producing cellulosomes and anaerobic bacteria without scaffoldin also exist.

In general, CBMs are appended to glycoside hydrolases that degrade insoluble polysaccharides. The main proposed roles of CBMs are to increase effective enzyme concentrations on the polysaccharide surface, to target the catalytic module to the substrate and eventually to disrupt the polysaccharide structure [7-9]. Traditionally, the presence of a functional CBM has been considered a requirement for full activity of cellulases on crystalline cellulose [7,10-12]. The CBMs of fungal CBHs all belong to CBM family 1 [5,13]. In GH-7 CBHs, the CBM is attached to the C-terminus, and in GH-6 enzymes, to the N-terminus via a flexible linker. The presence of CBMs has been shown to increase the concentration of protein on the surface of the substrate, and removal of the CBM from the cellulases or from the scaffoldin in the cellulosomes decreases dramatically the activity on insoluble, but not on soluble, substrates [14,15]. Recently, however, it has been shown that intact cellobiohydrolases and their core domains lacking CBM possess similar catalytic activity, *i.e.,* turnover number, towards cellulose [16], and that single molecules of both cellobiohydrolases (with and without CBM) proceed along the cellulose chain with a similar speed, implying that the presence of CBMs does not affect the turnover number [17]. These results indicate that the loading of a cellulose chain into the active site tunnel is also essential for the movement of an enzyme and can be achieved even without CBMs. The enzyme concentration, however, had to be raised to detect the adsorption of core domains on cellulose and to quantify the hydrolytic activity [17]. Hence, the CBM seems to have little, if any, effect on the actual catalysis and contributes primarily to the adsorption of enzymes to the substrate. CBMs have also been predicted to play a role in the processivity of cellobiohydrolase TrCel7A [18]. On the other hand, Kurasin and Väljamäe showed that the degree of processivity of cellobiohydrolases was limited by the length of obstacle-free path available on the cellulose chain [19], and thus proposed that enzymes capable of faster desorption would be needed to overcome the retardation of hydrolysis by cellulases being blocked by obstacles. Similar to adsorption, desorption is dependent on enzyme affinity to the substrate, and hence is strongly influenced by the presence of CBMs.

Apart from adsorption of cellulases on cellulose, CBMs also play a significant role in non-specific and non-productive adsorption on lignin, the major non-polysaccharide component of biomass, leading to a loss of enzyme activity during hydrolysis [20]. Prolonged contact of cellulases with lignin may also lead to irreversible denaturation of enzyme proteins, especially at elevated temperatures [21]. To prevent non-specific binding of cellulases on lignin, CBMs with different adsorption affinities can be engineered, or various chemicals, such as surface active agents, can be added to the hydrolysis [22]. In spite of numerous efforts, however, the yield of recyclable enzymes has remained low even with surfactants on lignin-rich substrates.

In this work we show the impact of substrate concentrations on the role of CBMs in the hydrolysis of insoluble substrates. Our hypothesis was that a high substrate concentration (or low water content of the system) would diminish the need for CBMs by bringing the catalytic entities into close physical association with the substrate. As model enzymes, we used the main well-characterized cellulases of the industrially important Ascomycete fungus *Trichoderma reesei,* with or without CBMs*.* Enzyme mixtures were designed by replacing the intact enzymes with core enzymes, one at a time or all together in the mixtures, composed to mimic the natural composition of *T. reesei* cellulases. All enzyme preparations were supplemented with β-glucosidase to prevent end product inhibition by cellobiose (Table 1).

**Table 1:** Cellulases and hemicellulases applied in the hydrolysis experiments

***Enzymes***			***Intact enzymes***		***Core enzymes***	
**New name**	**Old name**	**Loading**	**MW**	**Loading**	**MW**	**Loading**
		**μmol/g d.w.**	**kDa**	**mg/g d.w.**	**kDa**	**mg/g d.w.**
TrCel7A	CBH I	0.150	56.0	8.40	47.3	7.09
TrCel6A	CBH II	0.050	56.7	2.84	41.3	2.07
TrCel5A	EG I	0.025	51.9	1.30	42.9	1.07
TrCel7B	EG II	0.025	48.2	1.21	39.6	0.99
TrXyn11	Xylanase II	0.025	20.8	0.52	20.8	0.52
TrMan5A	Mannanase	0.010	47.9	0.48	41.4	0.41
AnCel3A	β-glucosidase	0.002	115.6	0.23	115.6	0.23
*Sum*				*14.50*^a,b^		*12.00*^a,b^

^a^ excluding mannanase.

^b^ In the hydrolysis experiments at various substrate consistencies, the total protein loadings equaled 14.5 and 15.0 mg/g d.w. (24.7 and 16.4 mg/g cellulose) intact enzymes and 12.0 and 12.4 mg/g d.w. (20.5 and 13.6 mg/g cellulose) core enzymes in the hydrolysis of wheat straw and Avicel, respectively.

Mannanase was added only to hydrolysis of Avicel.

## Results

### CBMs increase the efficiency of cellulases in diluted hydrolytic systems

**Figure 1:**
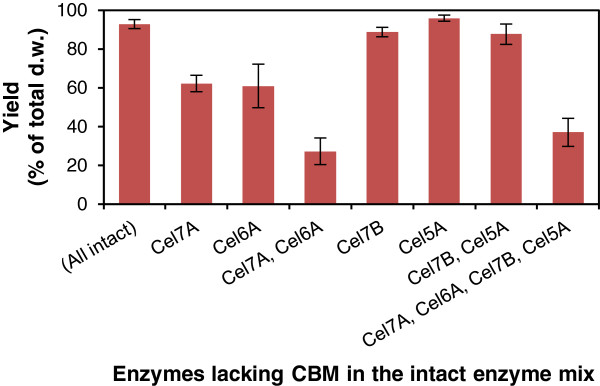
**Hydrolytic performance of *Trichoderma reesei *cellulases with and without their CBMs in enzyme mixtures.** Comparison of the hydrolytic performance of the *Trichoderma reesei* cellulases with and without their CBMs in enzyme mixtures on Avicel at a 1% (w/w) substrate consistency, after 48 hours at 45°C. In the enzyme mixture, the cellobiohydrolases Cel7A and Cel6A and endoglucanases Cel7B and Cel5A were replaced one-by-one, in pairs or all together with an equal molar amount of their core enzymes (Table 1).

CBMs are known to increase effective enzyme concentrations on the substrate surface and consequently, to enhance the performance of cellulose hydrolysis. Accordingly, both cellobiohydrolases TrCel7A and TrCel6A were clearly less efficient without CBMs than the intact enzymes in the hydrolysis of the cellulosic model substrate Avicel at a low substrate consistency of 1% (Figure 1). The excision of CBM resulted in a decrease in the overall hydrolysis yield from 93% to approximately 60% of the substrate (w/w) when one of the cellobiohydrolases was substituted by only its core domain and to 27% of the substrate when both cellobiohydrolases lacked CBMs. On the other hand, the endoglucanases TrCel5A and TrCel7B did not seem to lose their catalytic efficiency even without CBMs. Avicel was almost completely hydrolyzed irrespective of the presence of CBMs in the endoglucanases (Figure 1).

### Cellulases without CBMs catch up with cellulases with CBMs at elevated substrate concentration

**Figure 2:**
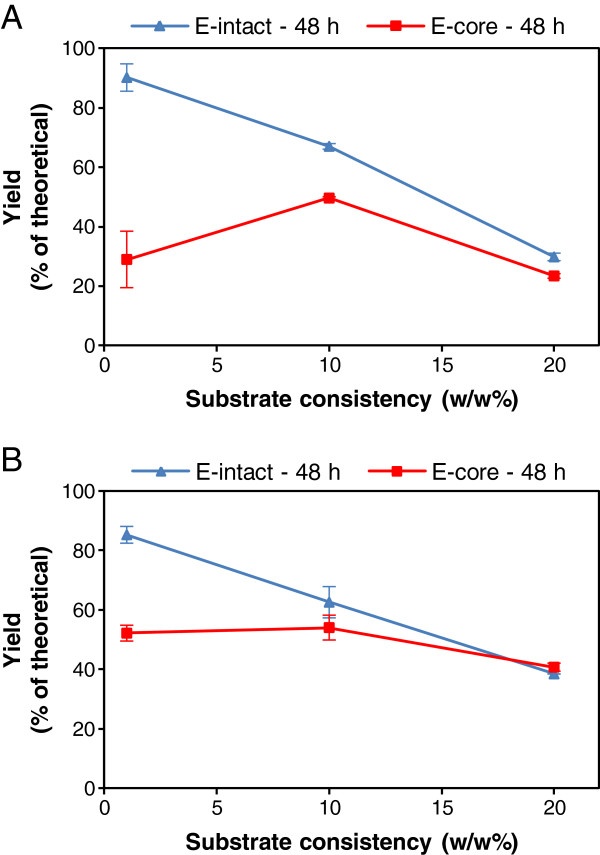
**Cellulases without CBMs catch up with cellulases with CBMs at elevated substrate concentration.** Hydrolysis yield as a function of substrate consistency after 48 hours hydrolysis of (**A**) Avicel and (**B**) pre-treated wheat straw at 45°C with the enzyme mixtures containing intact (blue) or core (red) enzymes (Table 1).

We compared the hydrolytic performance of a mixture of *T. reesei* enzymes composed of proteins, with or without CBMs, on natural substrates (Avicel and pre-treated wheat straw containing about 25% lignin) at low and high substrate consistencies, maintaining the ratio of enzyme to substrate constant. Hydrolysis of lignocellulose at elevated substrate concentrations leads to substantially increased absolute sugar concentrations, and the accumulation of hydrolysis products along with diffusion limitations causes more severe end-product inhibition of cellulases during hydrolysis. When increasing lignocellulose substrate concentration to approximately 25%, the hydrolysis yield, in general, is roughly halved [23]. Similarly, the hydrolysis yield of both substrates (pure cellulose and straw lignocellulose) decreased almost linearly from around 80-90% to approximately 30-40% of the theoretical yield by increasing substrate concentration in the system up to 20% (w/w) (Figure 2). Because of the lower affinity of the core enzymes to the substrate, the core enzymes lacking CBMs resulted in a lower hydrolysis yield than the intact enzymes at lower (1% and 10%) substrate consistencies (Figure 2). When increasing the concentration of both substrates, however, the difference in yield obtained with the mixtures of intact and core enzymes decreased gradually. At a 20% consistency of Avicel, the core enzymes were only slightly less efficient, while the wheat straw was hydrolyzed equally efficiently by both enzyme groups.

### Most of the cellulases without CBMs remain free throughout the hydrolysis

**Figure 3:**
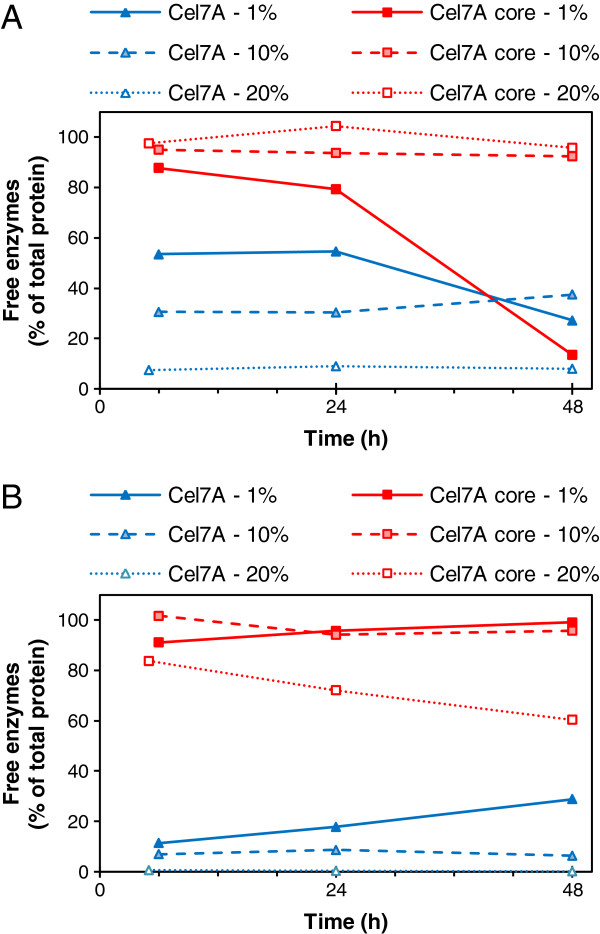
**The quantity of free TrCel7A throughout the hydrolysis.** Free intact (blue) and core (red) TrCel7A enzymes in the hydrolysates of (**A**) Avicel and (**B**) pre-treated wheat straw, identified with quantitative SDS-PAGE of the supernatants. The amount of free enzymes is expressed as% of the total TrCel7A enzyme load (core or intact) in the hydrolysis.

**Figure 4:**
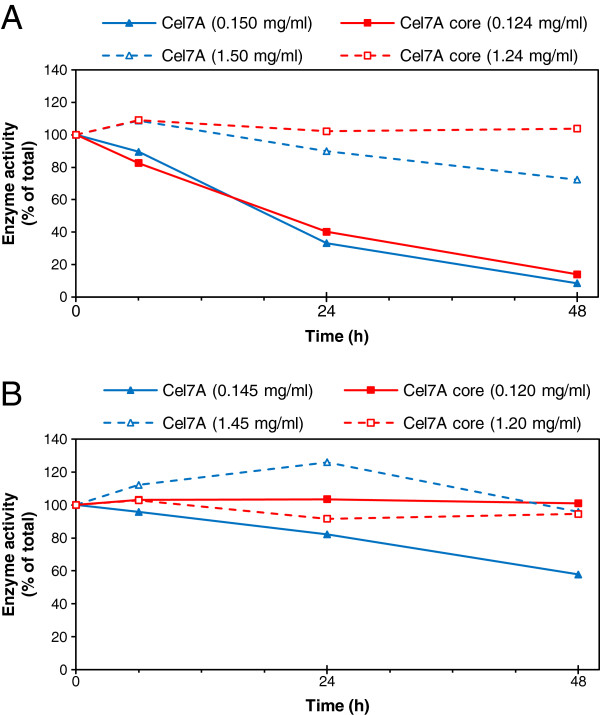
**Loss of activity of the main cellulase component, TrCel7A, when incubated at 45°C.** The activity of the intact (blue) and core (red) TrCel7A was measured against 4-methylumbelliferyl-β-D-lactoside as a function of time during incubation in **(A)** 50 mM citrate buffer (no protecting compounds present) and **(B)** wheat straw extract (wheat straw fraction soluble in the citrate buffer). The total protein loading of intact enzyme mixtures was **(A)** 0.150 and 1.50 mg/ml including mannanase, and **(B)** 0.145 and 1.45 mg/ml excluding mannanase, referring to hydrolysis experiments with 1% and 10% substrate consistency, respectively. Of the core enzyme mixtures, the total protein loading was **(A)** 0.124 and 1.24 mg/ml including mannanase and **(B)** 0.120 and 1.20 mg/ml excluding mannanase, corresponding to hydrolysis experiments at 1% and 10% substrate consistency, respectively.

During the hydrolysis of wheat straw with the core enzymes at lower consistencies (1% and 10%), more than 90% of the main enzyme component, TrCel7A core, could be detected in the supernatant, while the intact enzymes remained almost completely bound to the solid hydrolysis residue, especially at higher substrate consistencies. At 20% consistency, about 60% of the TrCel7A core could be recovered, while less than 2.5% of the intact TrCel7A remained free after 48 h hydrolysis of the wheat straw (Figure 3B) when reaching similar conversion in the hydrolysis. In the case of Avicel, a similar trend was observed: more than 90% of the core enzymes, while only 10% of the intact enzymes could be recovered (Figure 3A). The low recovery of core enzymes at a 1% (w/w) Avicel loading and the gradual decrease in the quantity of free enzymes during hydrolysis indicate enzyme inactivation in dilute solutions, which was confirmed by reference experiments on enzyme stability in the absence of substrate (Figure 4). The soluble fraction of wheat straw seemed to protect both the intact and core enzymes during hydrolysis (Figure 4B), while in the absence of this protecting fraction (in buffer), both the intact and core Cel7A lost 90% of their activity by the end of the incubation at a low protein concentration (Figure 4A). In the hydrolysis of Avicel or straw, the intact TrCel7A seemed to be protected due to adsorption by the CBM on the solid substrates. The recovery of individual free enzymes was estimated based on both quantitative SDS-PAGE and activity determinations, which gave equal results (Table 2). Most of the intact enzymes were adsorbed to a high extent when the substrate concentration was raised, whereas most of the core enzymes, especially the major protein Cel7A, could be recovered in the supernatant (Figure 3 and Table 2).

**Table 2:** Free cellulases in the hydrolysates after 48 h hydrolysis

**Substrate**	**Enzyme mix**	**Consistency**	**Enzyme recovery **^**a**^					**Cel7A recovery **^**a**^
		**% (w/V)**	**% of free protein of total applied**					**% activity in solution of total applied**
			*Cel7A*	*Cel7B*	*Cel6A*	*Cel5A*	*Cel3A*	*Based on MUL activity*
WS	Intact	1	29	61	13	19	110	21
		10	6	35	5	6	99	11
		20	bdl ^b^	8	6	bdl ^b^	4	3
	Core	1	99	100	64	90	131	85
		10	96	90	28	54	122	86
		20	60	16	0	18	23	59
A	Intact	1	27	38	23	23 ^c^	101	18
		10	38	60	30	26 ^c^	129	31
		20	8	15	11	bdl ^b,c^	97	6
	Core	1	14 ^d^	5 ^d^	6 ^c,d^	14 ^d^	68 ^d^	26 ^d^
		10	92	70	80 ^c^	100	127	65
		20	96	18	bdl ^b,c^	82	115	74

^a^ enzymes free in the supernatant after 48 h hydrolysis.

^b^ bdl = below detection limit.

^c^ enzyme and Man5A together (overlapping bands).

^d^ enzyme recovery was low due to enzyme inactivation.

Free cellulases in the hydrolysates after 48 h hydrolysis, separated and quantified by protein measurement by SDS-PAGE. The activity of Cel7A was also quantified in the supernatants using MUL as substrate.

**Figure 5:**
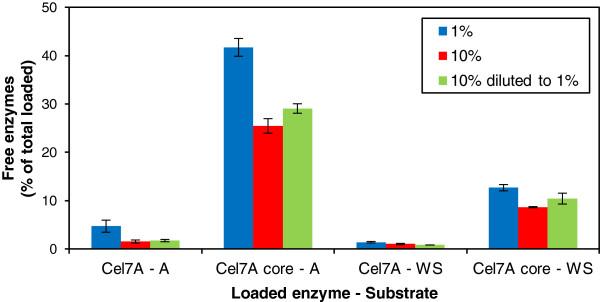
**Adsorption of intact and core TrCel7A on pre-treated wheat straw and Avicel.** Free intact or core Cel7A enzymes (as the percentage of total loaded enzymes) after incubation at 4°C in the presence of pre-treated wheat straw (WS) or Avicel (A) at 1% and 10% concentration (w/w). All samples were taken after 30 min when the equilibrium was reached. Additionally, pre-treated wheat straw (WS) and Avicel (A) were first incubated for 30 min at 4°C, then diluted tenfold and incubated for further 30 min before sampling (10% diluted to 1%) to study the reversibility of enzyme adsorption.

The aliquots of the hydrolysis experiments at 10% and 20% (w/w) substrate concentrations were at all points diluted tenfold prior to phase separation before analyzing enzymes and hydrolysis products in the supernatant in order to avoid inaccuracy of quantification [24]. Diluting the aliquots did not promote extensive desorption of enzymes as only 2-4% of the total loaded intact and core Cel7A enzymes could be desorbed from the solid substrate by diluting the aliquots (Figure 5). In fact, the adsorption of both intact and core Cel7A appeared to be only partially reversible. In addition, the adsorption of both intact and core enzymes was more pronounced at the higher substrate concentration (10% w/w) than at the lower substrate concentration (1% w/w). This supports the hypothesis of this work, *i.e.* that elevated substrate concentrations increase the possibility of enzyme-substrate interaction and hence improve the degree of hydrolysis.

### Occurrence of CBM in the nature

Today, surprisingly, many microorganisms are known to code cellulase genes without a CBM or dockerin module. The presence of the dockerin module implies that the enzymes would be a part of a cellulosome, containing CBMs. Analyzing the domain organization of cellulases in the Pfam database [25], which is comprised of available genomic data on amino acid sequences of enzymes produced by various microorganisms, revealed that most cellulases do not possess carbohydrate binding (or dockerin) modules (Table 3). Out of 717 identified or putative cellobiohydrolases belonging to GH family 7, only 94 sequences comprised sequence homology with that of CBM (family 1), and most of the putative cellulase protein sequences, *i.e.,* 623 sequences, lacked a binding module (Table 3). In general, less than 40% of the cellulases belonging to individual GH families contained a CBM. Analogously, Medie *et al.* and Eastwood *et al.* reported recently that only some glycoside hydrolases from bacteria or from genome sequenced fungi bear CBMs [26,27]. If the CBMs provided evolutionary advantages to cellulase-producing microorganisms (as cellulases with CBMs are generally accepted superior to cellulases lacking CBMs), why do most cellulases in nature seem to lack CBMs? Despite a few exceptions, such as GH 7 cellobiohydrolases of *Melanocarpus albomyces* and *Thermoascus aurantiacus,* most currently isolated and characterized cellobiohydrolases (EC 3.2.1.91 or 3.2.1.176) contain a CBM as listed in the Uniprot database [28]. Presumably, screening in diluted systems has led to the isolation of the most efficient enzymes under dilute test conditions, resulting in the choice of CBM-containing enzymes.

**Table 3:** Occurrence of carbohydrate binding modules (CBMs) in cellulases in the major glycoside hydrolase (GH) families

**GH family**	**Amino acid sequences containing CBM **^**a**^	**Amino acid sequences without CBM **^**a**^	**Reference entry**
	**All (Complete **^**b**^**)**	**All (Complete **^**b**^**)**	http://pfam.sanger.ac.uk/family/^**c**^
7	94 (89)	623 (234)	PF00840
6	176 (170)	263 (236)	PF01341
5	645 (597)	3192 (2617)	PF00150
12	31 (31)	354 (338)	PF01670
45	37 (37)	147 (122)	PF02015

^a^ CBM or dockerin implying that the enzyme is part of a cellulosome containing CBMs.

^b^ Amino acid sequences considered complete, *i.e.,* excluding fragments.

^c^ The straight link to the data is the combination of the Pfam Family link followed by the reference entry (i.e. the link to the Family 7 cellulases is http://pfam.sanger.ac.uk/family/PF00840, etc.).

Occurrence of carbohydrate binding modules (CBMs) in cellulases in the major glycoside hydrolase (GH) families based on amino acid sequences currently available in the Pfam database [25].

## Discussion

The generally recognized role of CBMs is to increase effective enzyme concentrations on the substrate surface and consequently, to enhance the performance of cellulose hydrolysis. Previously it was also shown that maximal hydrolysis rates (V_max_ values) did not remarkably decrease when omitting CBMs from TrCel7A and TrCel7B, whereas the K_E_ values were significantly increased, showing the obvious need to compensate for CBMs with a higher amount of enzymes [29]. This implies that the probability of collision between enzyme and substrate, and hence the hydrolysis rate, depends on the concentrations of enzymes. For an economically feasible hydrolysis process, however, the enzyme loading cannot be substantially raised. Therefore, we compared the hydrolytic performance of cellulases with or without CBMs at low and high substrate consistencies while maintaining the ratio of enzyme to substrate constant, which is equivalent to removing water from the system. Our results show that reducing the water content of the hydrolytic system (*i.e.* enabling close physical association and increasing the proximity of the enzymes and substrates in the system) promotes the adsorption of both intact and core enzymes to solid substrate, and hence seems to reduce the benefit of CBMs in dilute systems. As observed earlier [21,30], lignin impedes the hydrolysis of lignocellulose by adsorbing enzymes non-productively via CBMs. Consequently, the negligible difference in the performance of intact and core enzymes on pre-treated wheat straw at 20% (w/w) substrate loading could be the result of two parallel phenomena, namely enhanced enzyme-substrate interaction at high solid concentrations and reduced non-productive enzyme adsorption of the enzymes without CBMs on lignin.

Although most of the core enzymes could be recovered throughout the hydrolysis, similar hydrolysis yields were obtained with the bound intact and mostly free core enzymes (Figures 2 and 3). The intact cellulases, including the major protein Cel7A, seemed to be bound unproductively to the substrate to a higher extent than the core cellulases, as the core Cel7A performed the hydrolytic action equally effectively as the intact protein despite being adsorbed to a lower degree. When following the hydrolysis at 10% and 20% (w/w) substrate concentrations, the hydrolysis aliquots were at all points diluted prior to the phase separation for the analysis in order to avoid inaccuracy of calculating the amounts of components in the supernatant (enzymes and hydrolysis products) and evaluating hydrolysis yield. To evaluate hydrolysis at high concentrations of solid substrates, dilution of the whole suspension has been found to be the most reliable method [24]. The dilution of aliquots resulted in only a minor desorption of the intact and core Cel7A (2-4% of the total loading), and hence did not seem to promote enzyme desorption. In all conditions, a clear difference between the degree of binding of core and intact enzymes could be observed, leading to the possibility of recovering the core enzymes, especially the main protein Cel7A after hydrolysis. The possibility of enzyme recovery at higher solid consistencies is of high importance, potentially leading to significant reductions in enzyme costs for industrial-scale hydrolysis of biomass to platform sugars.

The conditions of high dry matter systems are similar to those of some of the natural habitats of lignocellulose-degrading fungi and bacteria on decaying wood. Pre-treated lignocellulosic substrates at solids concentrations above 25% (w/w) resemble mud-like wet soil, and practically no water (*i.e.* liquid phase) can be separated by centrifugation at 3000 rpm from the solid fraction. On the other hand, low dry matter systems are similar to aqueous environments such as hot springs. Therefore, it can be anticipated that environmental conditions, especially the amount of water in the natural habitat in which an organism (fungus or bacterium) lives, can be a major determinative factor in the evolutionary development of various microbial cellulolytic systems. The natural habitats of cellulase-producing microorganisms vary from aqueous (hot springs, animal rumen) to damp or wet (degrading composts and litter in soil) and to dry (rotting wood) environments. Unfortunately, no systematic study is currently available relating the origin or natural growth habitat of organisms with the characteristics of their cellulolytic systems. However, some rough correlations between the natural environment and cellulase systems can be emphasized based on the most extreme known cases. At one extreme, the aquatic anaerobic bacteria living in *e.g.* hot springs, intestines or other aqueous surroundings have evolved cell-bound cellulosomes because these organisms cannot afford to secrete free extracellular enzymes (or hydrolysis products) and have them taken away by the surrounding aqueous streams. Somewhat surprisingly, the presence of cellulosomes in anaerobic bacteria has not been discussed as a consequence of their aqueous habitat, which is the actual reason for their anaerobism. The product acquisition from the aqueous environment may be one further reason for the evolution of cellulosomes, as suggested by Gilbert *et al.*[6]. At the other extreme, some terrestrial fungi typically living on *e.g.* degrading wood logs may not gain such a benefit from CBMs, even though they secrete free cellulases into the surrounding area.

The majority of fungal cellulase producers so far characterized in detail belong to the phylum Ascomycota (127 of 148 strains producing GH7), which have variable growth habitats on degrading organic materials. Some ascomycetes such as *Trichoderma reesei* live on wet or very humid surroundings, such as degrading litter or compost, where CBMs could provide an advantage by recognizing and concentrating enzymes on the substrate. On the other hand, some other ascomycetes are reported to originate from fairly dry environments such as wood chips, saw dust or grain husks. For instance, the GH family 7 CBH, produced by *Sarcoscypha occidentalis*, which grows on decaying sticks and logs, does not contain a CBM. Some organisms such as aspergilli seem to encode enzymes of the same category (CBH) with and without a CBM [31]. In rumen microbiota, most cellulolytic microorganisms have been shown to bind tightly to cellulose, and neither secrete free cellulases nor produce cellulosomes [32]. Thus, organisms living in animal rumen (such as *Fibrobacter succinogenes*) may have developed an alternative mechanism for cellulose binding to the well-characterized (CBM-aided) hydrolytic systems. Thus, considerable variation concerning the presence of a CBM exists among various microbial species.

Our current knowledge of the role of CBMs is exclusively based on experiments carried out at low substrate concentrations, usually 0.1-2% (w/V). All published biochemical kinetic characteristics are based on determinations in dilute systems. However, no kinetic experiments have been performed at higher substrate concentrations where most of the biomass-degrading enzyme systems of various organisms are operational. The major obstacle in the practical performance of hydrolysis experiments at a high substrate concentration is the lack of techniques available, especially on a small laboratory scale. The common kinetic and practical hydrolysis tests used in screening for cellulases at low substrate concentrations could have produced data that may not apply for conditions resembling natural, low water-containing media. Thus, properties of many non-CBM containing enzymes screened for efficient hydrolysis may have been underestimated when tested in dilute conditions, leading to “throwing out the babies with the bath water.”

Undeniably, CBMs are important domains in the recognition of substrate and display significant specificity on various carbohydrate surfaces. It is clear that at low substrate concentrations, the probability is not high enough for the catalytic domain to recognize the substrate. The results presented here show, however, that the amount of water present could have important scientific and technical implications in the hydrolytic systems of enzymes, as it seems to determine the benefits of CBMs and to play a central role in the evolutionary development of various cellulolytic structures and systems. Reduction of water in industrial systems is a central economical aim to generate high final product concentrations. Technically, hydrolysis at high dry matter could potentially benefit from CBM-less enzymes by avoiding non-productive and irreversible binding and allowing reuse of enzymes.

## Methods

### Substrates

Avicel purchased from Serva was chosen as the model substrate of wood-derived microcrystalline cellulose, and washed insoluble fraction of hydrothermally pre-treated wheat straw (WS) pre-treated in Inbicon, Denmark, as the lignin containing, industrially available lignocellulosic raw material. The monosaccharide composition of the substrates was determined in a two-step hydrolysis with sulphuric acid according to the NREL-procedure [33] on a Dionex ICS-3000 gradient HPLC system (Dionex ICS-3000, Sunnyvale, CA) using CarboPac PA-1 column and 1 mL/min eluent flow and 30°C column temperature [34]. The Avicel contained 91.3% of dry weight (d.w.) cellulose and low amounts of both xylan (1.2%) and glucomannan (1.4%). The pre-treated wheat straw contained 58.6% of d.w. cellulose, 26.4% lignin, and 3.5% xylan. The lignin content of the wheat straw substrate was 26.4%.

### Enzymes

In the hydrolysis experiments, intact and truncated core enzymes lacking carbohydrate-binding modules (CBMs) from *Trichoderma reesei* were used: two cellobiohydrolases, TrCel7A and TrCel6A, two endoglucanases, TrCel5A and TrCel7B, and the mannanase TrMan5A as well as their catalytic domains. In addition, the xylanase TrXyn11 from *T. reesei* and the β-glucosidase AnCel3A from *Aspergillus niger*, both lacking CBMs, were supplemented to the enzyme mixtures. The intact cellulases, xylanase and mannanase were purified according to Suurnäkki *et al.*[35], Tenkanen *et al.*[36] and Stålbrand *et al.*[37], respectively, the truncated enzymes according to Suurnäkki *et al.*[35], and the truncated mannanase by a procedure modified slightly from the one used for TrCel5A. The β-glucosidase was purified according to Sipos *et al.*[38]. The protein content of the enzyme preparations was measured by the Bio-Rad DC (detergent compatible) Protein Assay based on the method of Lowry [39], and the molar mass of proteins by MALDI-ToF using a sinapic acid matrix with trifluoroacetic acid as the protonating agent.

### SDS-Page

The gel electrophoresis of the hydrolysates was performed with a Bio-Rad Criterion Stain Free Imager system to quantify the free enzyme components. The samples were mixed in a ratio of 3:1 with SDS-solution and boiled for 5 min, then loaded onto 10% Tris–HCl 1.0 mm Criterion Precast Gel and run in 25 mM Tris/192 mM glycine/0.1 M sodium dodecyl sulphate (SDS) buffer with 200 V and 100 mA for 55 min, using the Bio-Rad Precision Plus standard. The quantification was carried out with Image Lab software, as described previously [40].

The separation and order of the enzymes were checked by injecting individual enzymes into the wells of the gel. The bands of the intact cellulases followed (more or less) the order of the molecular weight measured by MALDI-ToF from the highest to the lowest as follows: Cel3A (115.6 kDa), Cel7A (56.0 kDa), Cel6A (56.7 kDa), Cel7B (51.9 kDa) and Cel5A (48.2 kDa) (Table 1). The core enzymes, according to their size, followed a different order: Cel3A (115.6 kDa), core Cel7A (47.3 kDa), core Cel7B (39.6 kDa), core Cel6A (41.3 kDa) and core Cel5A (42.9 kDa) (Table 1).

### Hydrolysis experiments

In the first hydrolysis experiment, Avicel was hydrolyzed at 45°C for 48 h at 1% (w/w) substrate concentration with the intact enzyme mixture (Table 1) and with enzyme mixtures where the cellobiohydrolases Cel7A and Cel6A and endoglucanases Cel7B and Cel5A were replaced one-by-one, in pairs or all together with an equal molar amount of the respective core enzymes. In the second experiment, both Avicel and pre-treated wheat straw were hydrolyzed at 45°C for 48 h with an enzyme mixture composed of either the intact or the core enzymes; all enzyme loadings were equal on a molar basis (Table 1). Mannanase was added only to Avicel, due to its mannan content. The total protein dosages equaled 14.5 and 15.0 mg/g d.w. (24.7 and 16.4 mg/g cellulose) intact enzymes and 12.0 and 12.4 mg/g d.w. (20.5 and 13.6 mg/g cellulose) core enzymes in the hydrolysis of pre-treated wheat straw and Avicel, respectively. The second hydrolysis was carried out at three different concentration levels, 1%, 10% and 20% (w/w), in total volumes of 2, 1 and 50 ml, respectively, at 45±2°C. The hydrolysis experiments at 1% and 10% (w/w) substrate concentrations were performed in tubes in water bath with magnetic stirring at 250 rpm using triplicates, and individual samples were withdrawn after 6, 24 and 48 hours. The hydrolysis experiment at 20% (w/w) substrate concentration was performed in duplicates with gravity mixing at approx. 60 rpm, and two samples were taken from each batch after 5, 24 and 48 hours. The hydrolysis aliquots at 10% and 20% (w/w) concentrations were diluted tenfold immediately prior to separating the solid and liquid phases (with centrifugation at 3000 rpm for 10 min) when sampling in order to minimize the measurement error introduced by the high amount of insoluble, leading to overestimation of the yield [24]. Using two different mixing systems did not affect the basic observation of this work.

For determination of enzymes in the supernatant, samples were taken prior to boiling. The free enzymes in the supernatant were quantified with SDS-PAGE, and the activity of cellobiohydrolase Cel7A was also measured using 4-methylumbelliferyl-β-D-lactoside (MUL) as substrate according to van Tilbeurgh *et al.*[41]. To determine the yield of released carbohydrates after hydrolysis, samples were boiled for 15 min and then analyzed for reducing sugars according to Miller [42].

### Enzyme inactivation

Reference experiments for the second hydrolysis experiment were carried out to estimate inactivation of enzymes during hydrolysis in the absence of substrates (Figure 4). For the Avicel reference, the enzyme mixtures were incubated in a 50 mM sodium-citrate buffer (pH 5.0). To mimic the effect of water-soluble components of wheat straw, wheat straw was extracted at 1 and 10% (w/w) concentrations with a 50 mM sodium-citrate buffer (pH 5) for 24 h at 45°C, and the liquid fraction separated by centrifugation was used in the enzyme stability test. The total protein loading of the intact enzyme mixtures including mannanase was set at 0.150 and 1.50 mg/ml (reference for Avicel hydrolysis, incubated with the citrate buffer), and excluding mannanase at 0.145 and 14.5 mg/ml (reference for wheat straw hydrolysis, incubated with the wheat straw extract), referring to hydrolysis experiments with 1% and 10% (w/w) substrate concentrations, respectively (Table 1). Of the core enzyme mixtures, the total protein loading was 0.124 and 1.24 mg/ml including mannanase, and 0.120 and 1.20 mg/ml excluding mannanase, referring to hydrolysis experiments with 1% and 10% substrate consistency, respectively.

### Enzyme adsorption and reversibility

Adsorption of intact and core Cel7A on pre-treated wheat straw and Avicel was determined at 4°C in a 50 mM sodium-citrate buffer (pH 5.0). Intact or core Cel7A was incubated in the presence of pre-treated wheat straw or Avicel at 1% or 10% (w/w) substrate loading. The enzyme loading corresponded to the dosage of Cel7A enzymes in the second hydrolysis experiment, *i.e.* 0.150 μmol/g d.w. (8.4 mg/g d.w. intact TrCel7A or 7.1 mg/g d.w. core TrCel7A), equaling 0.247 μmol/g cellulose of pre-treated wheat straw and 0.164 μmol/g cellulose of Avicel. After 30 min of incubation, the samples were filtered through a 0.45-μm-pore-size Acrodisc GHP syringe filter (Pall Corporation). In order to study the reversibility of adsorption, samples first incubated at 10% (w/w) substrate concentration for 30 min were diluted tenfold with 50 mM sodium-citrate buffer (pH 5.0), and further incubated for 30 min to obtain equilibrium before filtering (marked as 10% diluted to 1% in Figure 5).

## Conclusions

**Figure 6:**
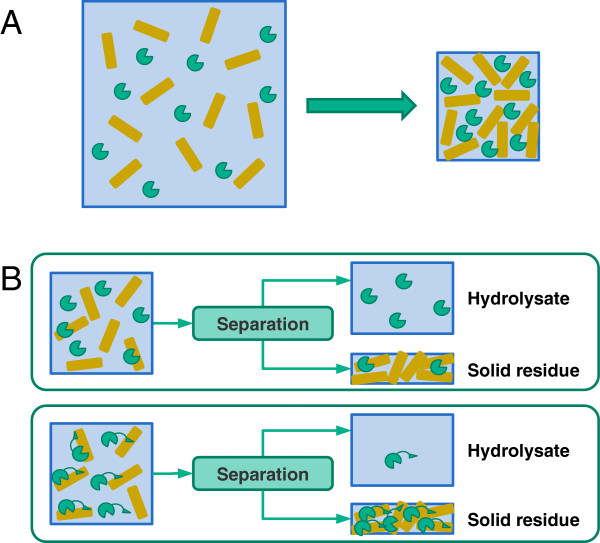
**Use of cellulases lacking carbohydrate-binding modules in hydrolysis at high substrate concentration.** (**A**) Reducing the amount of water helps enzymes (round shapes) find their substrates (brown bars). (**B**) Cellulases without CBMs (green round shapes) can be recovered after hydrolysis compared to intact cellulases (green round shapes joint by a triangle-shape unit representing CBMs).

Our hypothesis was that reducing the amount of water in hydrolysis could compensate for the absence of CBMs and lead to an equal hydrolytic efficiency with the intact and core enzymes. Accordingly, we clearly show that increasing the substrate concentration, i.e. decreasing the amount of water, helps enzymes find, recognize and adsorb onto the substrate surfaces even without CBMs (Figure 6A). Additionally, we also show that cellulases lacking CBMs were free in the hydrolysate after hydrolysis and could be available for reuse (Figure 6B). Our results confirm the hypothesis that the excision of CBMs resulted in an equally high degree of hydrolysis on both cellulosic and lignocellulosic substrates (Avicel and pretreated wheat straw) as the water content of the hydrolytic system was decreased. Presumably, the reduced non-productive adsorption of cellulases on lignin also had a positive effect on the hydrolysis at 20% substrate concentration with cellulases lacking CBMs.

Reducing the amount of water in hydrolytic systems favored the cellulases without CBMs. Therefore, it can be anticipated that environmental conditions, especially the amount of water in the natural habitat in which an organism (fungus or bacterium) lives, can be a major determinative factor in the evolutionary development of various microbial cellulolytic systems, e.g. with or without CBMs. Additionally, cellulases without CBMs could potentially help solve bottlenecks of enzymatic hydrolysis of lignocelluloses and speed up commercialization of second generation bioethanol.

## Abbreviations

CBH: Cellobiohydrolase;CBM: Carbohydrate-binding module;d.w: Dry weight;EG: Endoglucanase;GH: Glycoside hydrolase;HPLC: High performance liquid chromatography;MALDI-ToF: Matrix-assisted laser desorption/ionization time-of-flight;MUL: 4-methylumbelliferyl-β-D-lactoside;SDS-PAGE: Sodium dodecyl sulphate polyacrylamide gel electrophoresis

## Competing interest

The authors declare they have no competing interest.

## Authors’ contributions

AV., MS. and LV. designed the experiments. AV. performed the experiments and analyzed the data. All authors discussed the results and implications and commented on the manuscript at all stages. All authors read and approved the final manuscript.
